# Sirt6 regulates dendritic cell differentiation, maturation, and function

**DOI:** 10.18632/aging.100870

**Published:** 2016-01-12

**Authors:** Denise Lasigliè, Silvia Boero, Inga Bauer, Sara Morando, Patrizia Damonte, Michele Cea, Fiammetta Monacelli, Paizio Odetti, Alberto Ballestrero, Antonio Uccelli, Raul Mostoslavsky, Alessandro Poggi, Alessio Nencioni

**Affiliations:** ^1^ Department of Internal Medicine, University of Genoa, 16132 Genoa, Italy; ^2^ The Istituto di Ricovero e Cura a Carattere Scientifico Azienda Ospedaliera Universitaria San Martino-Istituto Scientifico Tumori, Istituto Nazionale per la Ricerca sul Cancro, 16132 Genoa, Italy; ^3^ Department of Neurology, Rehabilitation, Ophthalmology, Genetics, Maternal and Child Health, University of Genoa, 16100 Genoa, Italy; ^4^ The Massachusetts General Hospital Cancer Center, Harvard Medical School, Boston, MA 02114 USA

**Keywords:** Sirt6, dendritic cells, TNF-α, Toll-like receptor ligands, costimulatory molecules, immunosenescence

## Abstract

Dendritic cells (DCs) are antigen-presenting cells that critically influence decisions about immune activation or tolerance. Impaired DC function is at the core of common chronic disorders and contributes to reduce immunocompetence during aging. Knowledge on the mechanisms regulating DC generation and function is necessary to understand the immune system and to prevent disease and immunosenescence. Here we show that the sirtuin Sirt6, which was previously linked to healthspan promotion, stimulates the development of myeloid, conventional DCs (cDCs). Sirt6-knockout (Sirt6KO) mice exhibit low frequencies of bone marrow cDC precursors and low yields of bone marrow-derived cDCs compared to wild-type (WT) animals. Sirt6KO cDCs express lower levels of class II MHC, of costimulatory molecules, and of the chemokine receptor CCR7, and are less immunostimulatory compared to WT cDCs. Similar effects in terms of differentiation and immunostimulatory capacity were observed in human monocyte-derived DCs in response to SIRT6 inhibition. Finally, while Sirt6KO cDCs show an overall reduction in their ability to produce IL-12, TNF-α and IL-6 secretion varies dependent on the stimulus, being reduced in response to CpG, but increased in response to other Toll-like receptor ligands. In conclusion, Sirt6 plays a crucial role in cDC differentiation and function and reduced Sirt6 activity may contribute to immunosenescence.

## INTRODUCTION

Dendritic cells (DCs) are specialized sentinels of the immune system responsible for coordinating adaptive immune responses [1]. In addition, during the steady-state, DCs are also responsible for preventing aberrant immune activation, which is achieved through a tight regulation of DC environmental sensitivity, maturation, and life span. DCs arise from a hematopoietic lineage distinct from other leukocytes [2]. Two main DC subpopulations have been identified in so far: myeloid conventional DCs (cDCs), and plasmacytoid DCs (pDCs). cDCs exist in the peripheral tissues, in secondary lymphoid organs, and in the peripheral blood [1, 2]. cDCs have a dendritic shape and exhibit typical DC functions, such as antigen uptake, processing, and presentation. cDCs are largely deputed to IL-12 production and to the general orchestration of antigen-specific immune responses [2]. pDCs can be both of myeloid and lymphoid origin and they are primarily in charge for secreting type I interferons in response to viruses [1, 2].

Impaired DC function is a hallmark of many types of disorders, including cancer [3], and is commonly observed during aging, too, contributing to reduce immune competence, but also predisposing to inflammation through aberrant cytokine production (i.e. IL-6 and TNF-α) [4-6]. Defining the mechanisms that underlie DC lineage commitment, differentiation, maturation and function represents a key challenge which will improve our understanding of the balance between immunity and tolerance and possibly also lead to new strategies to boost immune competence and avoid unwanted inflammation.

The NAD^+^-dependent deacetylase Sirt6, a protein involved in genome maintenance and in metabolic homeostasis, exerts protective functions against age-related diseases and was shown to extend life span in mice [7-9]. In turn, a decline in Sirt6 expression [10, 11] and/or in its function (as a result of reduced availability of tissue NAD^+^) [12] are postulated to contribute to human aging [8]. Quite remarkably, some of Sirt6's protective roles appear to reflect a role in immune regulation for it. Specifically, Sirt6 contributes to the release of TNF-α, a cytokine that is critically required for immune competence, via direct deacylation of TNF-α itself [2, 13-15]. Data from our laboratory indicate that Sirt6 also favours the secretion of IFN-γ and of the pro-chemotactic cytokine CXCL8 via *O*-acetyl-ADP-ribose production and consequent activation of the TRPM2 cation channel, and of Ca^2+^ signalling downstream of it [13, 16, 17]. In addition, a role for Sirt6 in the resolution of inflammation has also been proposed based on the detection of inflammatory infiltrates in tissues from Sirt6 knockout (Sirt6KO) mice [9, 18] and on the ability of Sirt6 to reduce NF-κB and c-JUN activity [18, 19] as well as glycolysis in activated T cells [20].

Sirt6 is expressed in bone marrow (BM)-derived cDCs (BMDCs) [15]. Nevertheless, the contribution of this enzyme to the biology of this important leukocyte subset remains presently unknown. Taking advantage of the availability of Sirt6KO mice [9], we focused on the role of Sirt6 in the differentiation, maturation and function of BMDCs generated with granulocyte-macrophage colony-stimulating factor (GM-CSF) [21, 22], and, making use of a chemical SIRT6 inhibitor [23], we validated our findings in human monocyte-derived DCs (moDCs). We identify a novel, key role for Sirt6 in cDC biology.

## RESULTS

### Sirt6 promotes cDC lineage commitment

**Figure 1:**
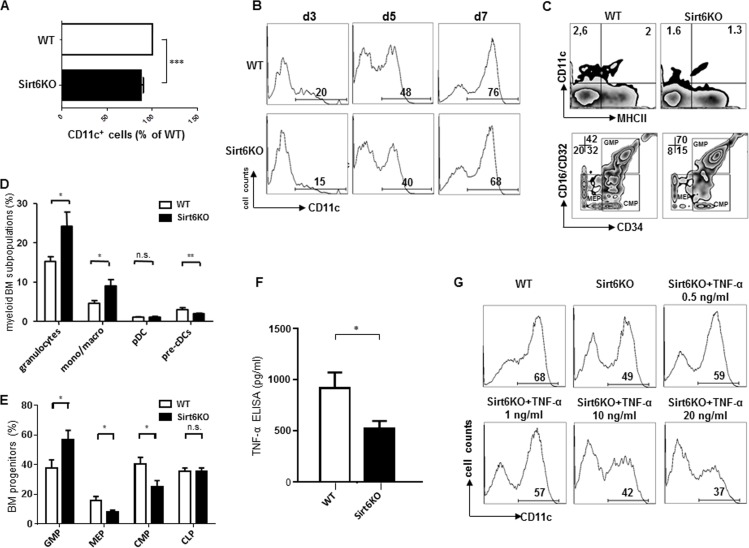
Sirt6 regulates the generation of cDCs *in vivo* and *in vitro* (**A**, **B**) WT or Sirt6KO BM cells were cultured with GM-CSF and their ability to generate CD11c^+^ cells was analyzed at day 3, 5, 7 by flow cytometry. (**A**) The percentage of CD11c^+^ cells within cultures of Sirt6KO BMs (day 7) was normalized to that of WT CD11c^+^ cells. Results are presented are means ± SEM of 6 separate experiments, n=13 for each genotype; ***: p<0.001. (**B**) one representative experiment out of two is presented. (**C**-**E**) BM cells from WT and Sirt6KO mice were analyzed by flow cytometry for the frequency of cDC precursors (pre-cDCs, CD11c^+^MHCII^−^), pDCs, monocyte/macrophage subsets, mature granulocytes and of BM progenitors of different lineages. (**C**) one representative experiment out of six is presented; (**D**, **E**) results are presented are means ± SEM of fifteen and six separate experiments, respectively, n=6-15 for each genotype; *: p<0.05; **: p<0.01; n.s.: not significant. (**F**) TNF-α concentration in the supernatants of WT and Sirt6KO BMDCs (harvested at day 6) were determined by ELISA. Results are means ± SEM of three separate experiments, n=10 for each genotype; *: p<0.05. (**G**) WT and Sirt6KO BM cells were cultured with GM-SCF with or without addition of the indicated concentrations of TNF-α. CD11c^+^ cells were quantified at day 6 by flow cytometry. One representative experiment out of six is presented, n=6-9 for each genotype.

Culturing mouse BM in the presence of GM-CSF typically results in the generation of large amounts of CD11c^+^ BMDCs that, by day 8 of culture, reach a 70% purity and typically comprise a mixture of immature and mature BMDCs [21, 22]. Thus, this model was first used to compare BMDCs from wild type (WT) and Sirt6KO mice and thereby assess a potential role for Sirt6 in cDC development. The yield of CD11c^+^ BMDCs from BMs harvested from Sirt6KO mice was typically lower than that obtained with WT BMs (Figure 1A). Time course experiments demonstrated how already at early time points the rates of CD11c^+^ BM cells cultured with GM-CSF were lower when Sirt6 was missing (Figure 1B), suggesting that the observed difference may reflect, at least in part, a reduced representation of the myeloid cDCs lineage in the BM of Sirt6KO mice. Consistent with this hypothesis, an *ex vivo* analysis showed a significant reduction in CD11c^+^/MHCII^−^ cells (cDC precursors, pre-cDCs) in the BM of Sirt6KO mice as compared to the control mice (Figure 1C, D). Quite remarkably, Sirt6 deficiency had no effect on the representation of pDCs in the BM (Figure 1D), while other mature myeloid subsets, such as granulocytes and mono/macrophages were even more represented in Sirt6KO BM than in WT BM. Studies of early myeloid precursors demonstrated a reduced frequency of common myeloid progenitors (CMP) and of megakaryocyte-erythroid progenitors (MEP) in Sirt6KO BM in favor of an expansion of granulocyte macrophage progenitors (GMP) (Figure 1E), the latter finding being consistent with the detection of increased frequencies of granulocytes and macrophages in Sirt6KO BM (Figure 1D). Notably, peripheral blood hematology performed in animals aged 17-18 days revealed no effect of Sirt6 deletion on total white blood cell counts (which, however, did not discriminate between the different white blood cell subsets), while a reduced mean corpuscular hemoglobin concentration as well as reduced platelets counts in Sirt6KO mice could be documented (Supplementary Table 1). Overall, the previous experiments demonstrated that Sirt6 deletion reduces cDC lineage commitment and that a reduced frequency of CMP and of cDC precursors is likely to contribute to the reduced yield of CD11c^+^ BMDCs observed *in vitro* with Sirt6KO BM.

TNF-α is a pleiotropic cytokine whose role in regulating hematopoiesis and cDC generation has been documented in both human and mice [24, 25]. In turn, Sirt6 was previously reported to promote TNF-α secretion [13, 15, 16]. Indeed, consistent with these studies, reduced TNF-α concentrations were detected in BMDC supernatants from Sirt6KO mice compared to WT BMDCs (Figure 1F). Thus, we hypothesized that a reduced TNF-α availability in Sirt6KO mice could explain the lower numbers of cDC precursors observed in the BM and also contribute to the inefficient *in vitro* generation of BMDCs. To test the latter hypothesis, different concentrations of recombinant mouse TNF-α were added to the cultures of differentiating Sirt6KO BMDCs and the frequency of CD11c^+^ cells was analyzed and compared to that of WT BMDCs. Supplementation with concentrations of TNF-α that mimicked those found in the supernatants of WT BMDCs (0.5-1 ng/ml) was indeed found to increase the frequency of CD11c^+^ cells in Sirt6KO BMs (p<0.05; n=9 for each genotype) without, however, fully reverting the original phenotype (Figure 1G).

Interestingly, higher TNF-α concentrations (10-20 ng/ml) not only failed to increase the frequency of CD11c^+^ BMDCs in cultured Sirt6KO BMs, but they even reduced it (p<0.01 for 10 ng/ml TNF-α, and p<0.001 for 20 ng/ml TNF-α; n=6 for each genotype). Thus, overall, these experiments indicated that Sirt6 promotes BMDC differentiation in a way that is partially dependent on its ability to promote TNF-α secretion.

### Sirt6 deficiency prevents the spontaneous maturation of *in vitro* generated BMDCs in a partially TNF-α dependent fashion

**Figure 2:**
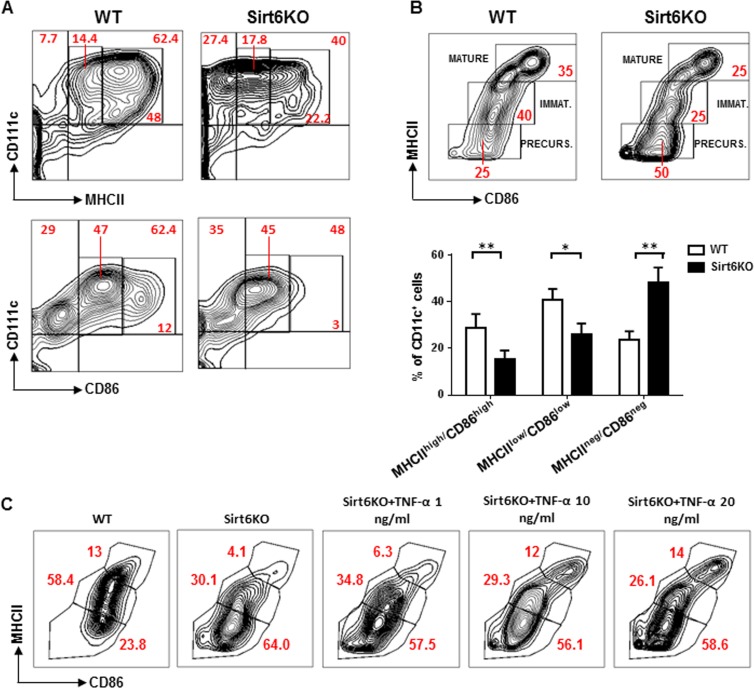
Sirt6 deletion hampers the spontaneous maturation of BMDCs (**A**, **B**) BM cells from WT and Sirt6KO mice were cultured for 6 days with 20 ng/ml GM-CSF. At day 6, cells were re-seeded in the presence of 5 ng/ml GM-CSF and CD86, MHCII, and CD11c expression was determined at day 7 by flow cytometry. (**A**, **B**) One representative experiment out of eight is presented. (**B**) lower inset, results are means ± SEM of eight separate experiments; n=13 for each genotype; *: p<0.05; **: p<0.01. (**C**) WT and Sirt6KO BMDCs harvested at day 6 of culture were re-seeded with TNF-α at the indicated concentrations. 24 h later, BMDCs were harvested and analyzed by flow cytometry. One representative experiment out of five is presented (n=8 for each genotype).

Subsequent experiments were directed at defining the phenotypic and functional features of Sirt6KO BMDCs, starting from their degree of maturation. Indeed, BMDCs generated *in vitro* with GM-CSF undergo a spontaneous maturation process that is characterized by the expression of different levels of MHCII and costimulatory molecules CD80 (B7.1) and CD86 (B7.2), depending on the degree of maturation reached [21]. High levels of MHCII and of CD80 and CD86 are considered hallmarks of mature BMDCs, while immature BMDCs and BMDC precursors are characterized by low and no MHCII expression, respectively [21]. As compared to WT BMDCs, Sirt6KO BMDCs were found to express lower levels of MHCII, CD86 (Figure 2A) and CD80 (see below), while class I MHC expression was not affected (data not shown). When using MHCII and CD86 expression to distinguish between mature BMDCs (CD11c^+^/MHCII^high^/CD86^high^), immature BMDCs (CD11c^+^/MHCII^low^/CD86^low/neg^), and BMDC precursors (CD11c^+^/MHCII^−^/CD86^−^), in Sirt6KO BMs cultured in the presence of GM-CSF, we detected a clear decrease in the representation of both mature and immature BMDCs. This effect was accompanied by an accumulation of BMDC precursors in Sirt6KO BM cultures as compared to cultures of WT BM (Figure 2B). Thus, these data indicate that Sirt6 deletion impairs the spontaneous maturation process that BMDCs normally undergo.

The role of TNF-α in cDC differentiation and maturation has been largely described, both in mice and humans [21, 26]. To test whether the impaired differentiation/maturation of Sirt6KO BMDCs would reflect their reduced ability to secrete TNF-α (Figure 1F), we added different amounts of this cytokine to the cell cultures at 6 day and evaluated the maturation achieved by Sirt6KO and WT BMDCs 48 h later. As shown in Figure 2C, adding TNF-α at concentrations that mimicked those found in cultures from WT BMDCs (i.e. 1 ng/ml) was not sufficient to increase the frequency of Sirt6KO mature BMDCs to the control levels (WT mature BMDC vs. Sirt6KO mature BMDC: p<0.01; n=8 for each genotype). However, higher TNF-α concentrations did increase the representation of mature BMDCs to the levels observed within WT BMDCs (p=0.49 for WT vs. Sirt6KO BMDCs plus 10 ng/ml TNF-α, and p=0.32 for WT vs. Sirt6KO BMDCs plus 20 ng/ml TNF-α; n=8 for each genotype). 1 ng/ml and 10 ng/ml TNF-α failed to increase the frequency of immature (CD11c^+^/MHCII^low^/CD86^low/neg^) Sirt6KO BMDCs to the control level (p=0,0373 and p=0.0151 vs. WT immature BMDCs, respectively), while TNF-α at a concentration of 20 ng/ml even further reduced the representation of immature BMDCs in cultured Sirt6KO BM cells (p<0.001). Thus, overall, the defective spontaneous maturation that Sirt6KO BMDCs exhibit appears to partially reflect their reduced production of TNF-α. However, the contribution of other mechanisms cannot be excluded.

**Figure 3:**
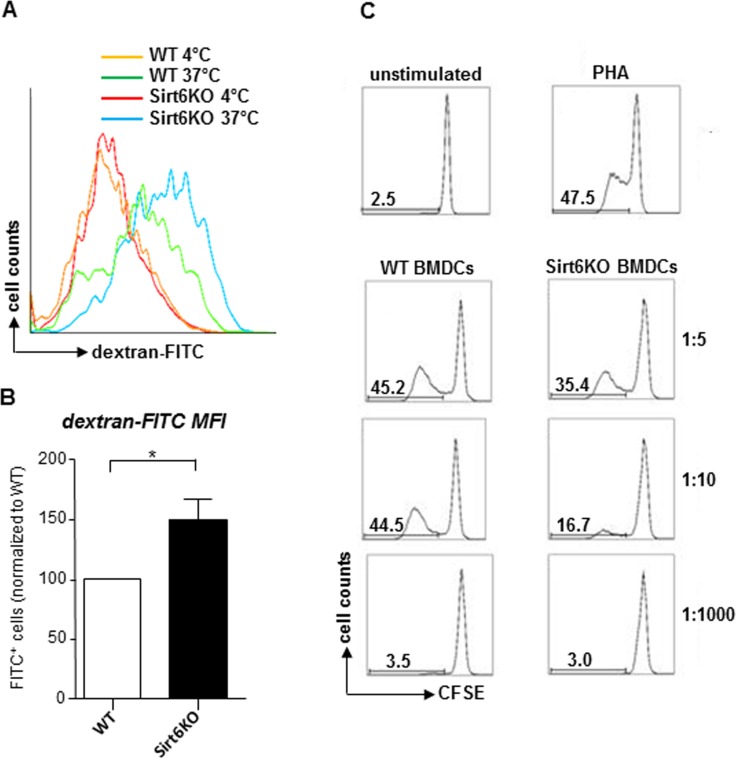
*In vitro* generated Sirt6KO BMDCs show increased endocytic activity and impaired allostimulatory capacity (**A**, **B**) WT and Sirt6KO BMDCs were harvested at day 7 and incubated with dextran-FITC for 30 min at 37°C or at 4°C. Thereafter, cells were stained for CD11c and finally analyzed by flow cytometry. (**A**) Gating was done on CD11c^+^ cells; one representative experiment out of six is presented. (**B**) Dextran-FITC^+^/CD11c^+^ Sirt6KO BMDCs were enumerated and their frequency was normalized to that of dextran-FITC^+^/CD11c^+^ WT BMDCs. Results are means ± SEM of six separate experiments, n=6 for each genotype. (**C**) purified allogeneic (BALB-c) CD4^+^ splenocytes (responders) were stained with CFSE and incubated with 5 μg/ml phythoemagglutinin (PHA) or with sorted, WT or Sirt6KO, CD11c^+^MHCII^+^ BMDCs at the indicated S:R ratios. Proliferation of alive (propidium-iodide negative) CD3^+^CD4^+^CD11c^−^ cells was evaluated by carboxyfluorescein succinimidyl ester (CFSE) dilution after a 5-day incubation. One representative experiment out of three is presented, n=4 for each genotype.

cDC maturation is typically accompanied by a loss in their ability to capture antigens and by an increase in their immunostimulatory capacity [26]. Consistent with a highly immature phenotype, Sirt6KO BMDCs were found to exhibit an increased endocytic activity compared to the WT BMDCs, as detected by monitoring FITC-dextran uptake (Figure 3A, B). A more detailed analysis demonstrated that the Sirt6KO BMDCs that exhibited increased dextran uptake were primarily the CD11^+^/MHCII^−^ precursors and, interestingly, also the mature (CD11^+^/MHCII^high^) BMDCs, while immature (CD11^+^/MHCII^low^) Sirt6KO BMDCs essentially presented an uptake that was comparable to the WT BMDCs (Supplementary Figure 1). Finally, in line with a less mature phenotype, Sirt6KO BMDCs also showed a reduced ability to simulate allogeneic lymphocyte proliferation in mixed leukocyte reaction (MLR;, Figure 3C; p<0.05 at a stimulator-to-responder - S:R - ratio of 1:10; n=4 for each genotype).

### Sirt6 contributes to BMDC maturation in response to Toll-like receptor ligands

**Figure 4:**
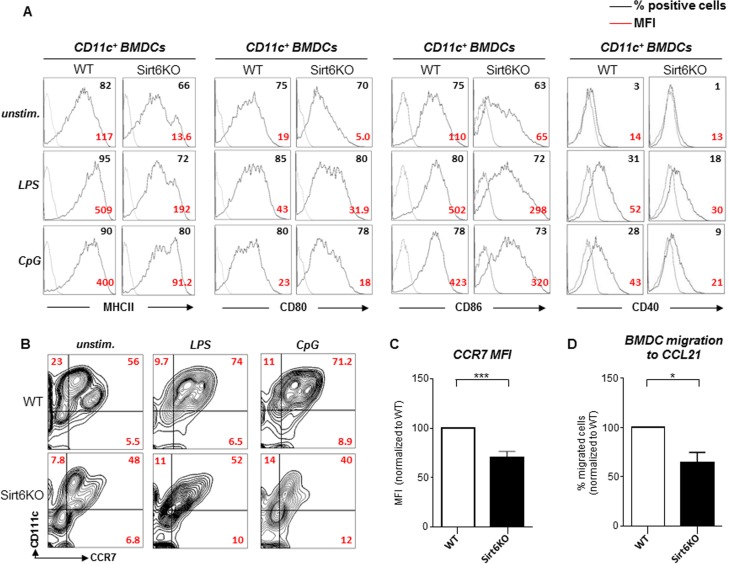
Sirt6KO BMDCs show impaired maturation and CCR7 expression in response to TLR ligands (**A**-**C**) WT and Sirt6KO BMDCs were harvested at day 7 and stimulated with or without LPS or with CpG for 24 h. Thereafter, cells were harvested, washed, and MHCII, CD86, CD80, CD40 and CCR7 expression on CD11c^+^ cells was analyzed by flow cytometry. (**A**, **B**) One representative experiment out of ten (**A**) or out of four (**B**) is presented (n=4-21 for each genotype). (**C**) CCR7 mean fluorescence intensity (MFI) was normalized to that of WT BMDCs. Results are means ± SEM of four separate experiments, n=11 for each genotype; ***: p<0.001. (**D**) WT and Sirt6KO BMDCs were harvested at day 7 and stimulated for 24 h with LPS. Thereafter, cell migration to CCL21 was evaluated. The percentage of Sirt6KO BMDCs that had migrated was normalized to that of WT BMDCs. Results are means ± SEM of three separate experiments, n=4 for each genotype; *: p<0.05.

The initiation of adaptive immune responses is critically dependent on cDC maturation. Complete cDC maturation is typically triggered by conserved molecular patterns that are expressed by infectious agents and that bind Toll-like receptors (TLRs) on immune cells, or by pro-inflammatory cytokines [1, 2]. To test the consequences of the absence of Sirt6 on BMDC maturation in response to TLR ligands, we stimulated WT and Sirt6KO BMDCs with lipopolysaccharide (LPS) or CpG oligonucleotide (CpG) and then monitored the expression of MHCII, CD80, CD86, as well as of CD40, a key costimulatory molecule involved in cDC licensing by activated T cells [27]. As previously mentioned, in the absence of stimuli, Sirt6KO BMDCs were found to express lower levels of MHCII, CD80 and CD86 as compared to WT BMDCs, while CD40 expression was essentially undetectable in either type of BMDCs (WT and Sirt6KO) (Figure 4A). Following stimulation with LPS or CpG, both WT and Sirt6KO BMDCs upregulated all of the surface markers. Nevertheless, MHCII, CD80, CD86, and CD40 expression on Sirt6KO BMDCs was consistently lower as compared to the WT BMDCs regardless of the stimulus that was applied. Prolonging the stimulation or increasing the concentration of the stimuli failed to abrogate this difference (data not shown). Notably, quantitative PCR (qPCR) failed to identify significant differences between WT and Sirt6KO BMDCs in terms of expression of *Tlr4* (LPS receptor) and *Tlr9* (CpG receptor), as well as of *Tlr2*, *Tlr3*, and *Tlr7* (Supplementary Figure 2A), indicating that the defective maturation of Sirt6KO BMDCs in response to the TLR ligands is not due to reduced expression of the TLRs themselves. Overall, these data clearly indicate that Sirt6 takes part in final maturation of BMDCs in response to TLR ligands and that Sirt6 deficiency results in a lower expression of MHCII and of costimulatory molecules on maturing cDCs.

Mature BMDCs also upregulate the CC-chemokine receptor 7 (CCR7), which, in turn, allows cDC migration to the lymph nodes via afferent lymphatics [1]. Specifically, CCR7-dependet migration occurs in response to ligands such as CCL21. Once again, consistent with a lower degree of maturation, both unstimulated and LPS- or CpG-stimulated Sirt6KO BMDCs exhibited reduced CCR7 expression as compared to the WT BMDCs (Figure 4B, C). Furthermore, in line with a reduced CCR7 expression, CCL21-driven migration of LPS-stimulated Sirt6KO BMDCs was found to be defective compared to the control WT BMDCs (Figure 4D). A similar impairment in CCL21-driven migration was also observed in Sirt6KO BMDCs that had been stimulated with CpG or with polyinosinic:polycytidylic acid (poly:IC; data not shown).

### Sirt6 deletion skews cytokine production by BMDCs

**Figure 5:**
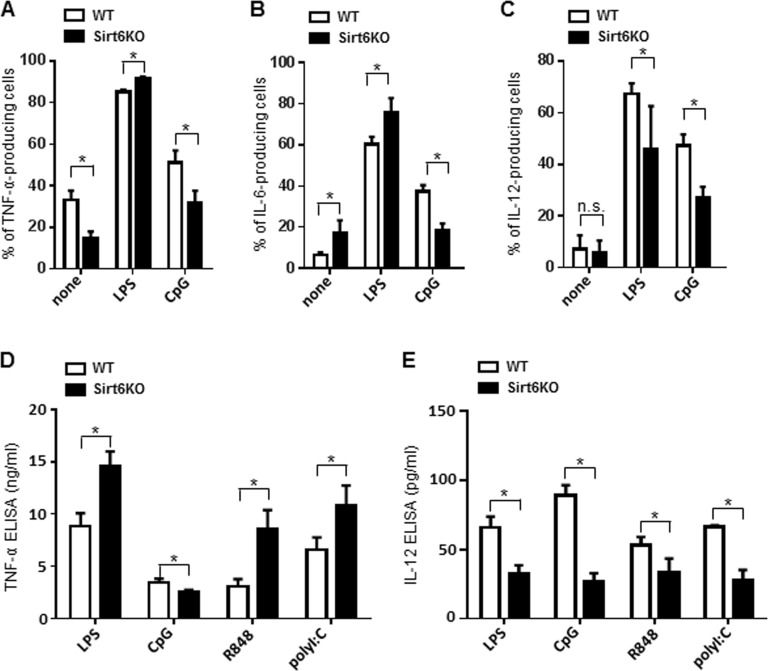
Sirt6 deletion skews cytokine production in BMDCs (**A**-**E**) WT and Sirt6KO BMDCs were harvested at day 8 and stimulated for 24 h with or without different TLR ligands. (**A**-**C**) Cells were harvested, stained for CD11c, CD86 and intracellular TNF-α, IL-6 or IL-12. CD11c^+^CD86^+^ TNF-α-, IL-6-, or IL-12-producing cells were quantified by flow cytometry. Results are means ± SEM of three-to-five separate experiments, n=3-10 for each genotype. In (**D**, **E**), cytokine secretion into cell supernatants was measured by ELISA. Results are means ± SEM of 3-10 separate experiments; *: p<0.05; n.s.: not significant.

Mature cDCs normally secrete large amounts of cytokines which, in turn, contribute to the overall orchestration of an effective immune response [1, 2]. We used intracellular cytokine staining and ELISAs to monitor the effects of Sirt6 deficiency on the ability of BMDCs to produce TNF-α, IL-6 and IL-12. Using these approaches, in Sirt6KO BMDCs, we found a general reduction in the expression of most of the cytokines analyzed. In unstimulated Sirt6KO BMDCs, there was a decrease in the percentage of CD11c^+^CD86^+^ TNF-α-producing cells (Figure 5A, Supplementary Figure 3A) and TNF-α concentration in cell supernatants was also consistently reduced (919 ± 154.6 pg/ml in WT BMDCs, n=7; 523 pg/ml ± 76.6 pg/ml in Sirt6KO BMDCs, n=10; p<0.05). Interestingly, in the absence of stimuli, the expression of IL-6 in Sirt6KO BMDCs was significantly increased both in terms of frequency of CD11c^+^CD86^+^ IL-6-producing cells (Figure 5B, Supplementary Figure 3B) and of IL-6 concentration in cell supernatants (248 pg/ml ± 56.8 in WT BMDCs, n=3; 506,7 ± 51,8 pg/ml in Sirt6KO BMDCs, n=3; p<0.05). However, upon BMDC stimulation with TLR ligands, we observed different and stimulus-dependent cytokine expression patterns. In response to a 24-h stimulation with LPS, there was a higher frequency of CD11c^+^CD86^+^ TNF-α-producing cells among the Sirt6KO BMDCs as compared with the controls (Figure 5A, Supplementary Figure 3A), which was accompanied by a consistent increase in TNF-α secretion (Figure 5D). The same effect was observed in response to the TLR2 ligand zymosan (Zym; TNF-α concentrations in the supernatants of Zym-stimulated BMDCs were 56.9 ± 21.6 ng/ml for WT cells, n=5; and 91.4 ± 14.5 ng/ml for Sirt6KO cells, n=5; p<0.05). As already documented elsewhere [15, 16], CpG was less effective at increasing the frequency of CD11c^+^CD86^+^ TNF-α-producing cells (Figure 5A, Supplementary Figure 3A) and at stimulating TNF-α secretion (Figure 5D) in Sirt6KO BMDCs than it was in WT BMDCs. Notably, a reduced TNF-α production by Sirt6KO BMDCs appeared to be recorded in response to CpG, but not to ligands of other endosomal TLRs: stimulating TLR7/8 with R848 resulted in higher frequencies of CD11c^+^CD86^+^ TNF-α producing cells among the Sirt6KO BMDCs (80.5 ± 1,7% TNF-α-producing WT BMDCs, n=9; 85.0 ± 1.2% TNF-α-producing Sirt6KO BMDCs, n=9; p<0.05) and in a consistent increase in TNF-α secretion into cell supernatants by the latter as compared to the control BMDCs (Figure 5D). Similarly, the frequency of CD11c^+^CD86^+^ TNF-α-producing BMDCs in response to the TLR3 ligand, polyI:C, was higher among the Sirt6KO BMDCs as compared to the WT BMDCs (92% ± 1,6% vs. 84 ± 1.5%, respectively; p<0.05) and a similar pattern of TNF-α secretion was observed in cell supernatants (Figure 5D). As previously anticipated, the reduced ability of CpG to stimulate TNF-α (as well as IL-6, see below) production in Sirt6KO BMDCs did not reflect a reduced expression of its receptor (*Tlr9*) (Supplementary Figure 2A) and neither was it associated with an impaired expression of *Unc93b1*, a protein involved in the trafficking and proper function of endosomal TLRs (Supplementary Figure 2B) [28, 29]. Incubating the BMDCs for shorterperiods of time (2h, 4h, 6h) or increasing the concentration of TLR ligands up to 10 fold essentially did not modify these differences (data not shown). The effect of Sirt6 deletion on the frequency of CD11c^+^CD86^+^ IL-6-producing BMDCs and on IL-6 secretion into cell supernatants in response to the different TLR ligands resembled that observed for TNF-α (Figure 5B, Supplementary Figure 3B and data not shown). On the other hand, IL-12 exhibited a completely different pattern of expression between WT and Sirt6KO BMDCs. Namely, in absence of Sirt6, we detected a strong reduction in the frequency of IL-12p70-producing BMDCs, as well as in IL-12 concentration in BMDCs supernatants, in response to all of the TLR ligands tested (Figure 5C, E, Supplementary Figure 3C). These data highlight for the first time a role for Sirt6 in the control of IL-12 production and strengthen the notion that the contribution of Sirt6 to the regulation of cytokine expression is both stimulus- and cytokine-dependent.

### SIRT6 inhibition prevents moDC differentiation

**Figure 6:**
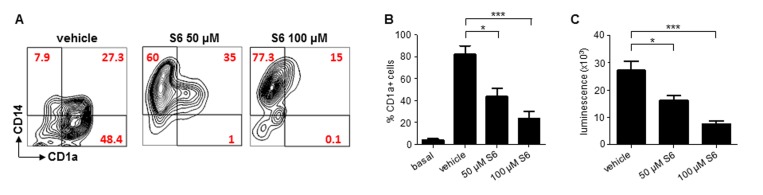
SIRT6 inhibition prevents moDC differentiation (**A**-**C**) Human adherent PBMCs were cultured with GM-CSF and IL-4 for 6 days in the presence of either S6 (at the indicated concentrations) or vehicle DMSO. Thereafter, cells were harvested, washed and analyzed by flow cytometry for CD14 and CD1a expression or utilized as stimulators in MLR. (**A**) One representative experiment out of five is presented. (**B**, **C**) Results are means ± SEM of those obtained with five different donors. *: p<0.05; ***: p<0.001.

Having established that Sirt6 plays a critical role in BMDC differentiation, maturation and function, we aimed at assessing whether such a role would be present in human cDCs, too. moDCs are a broadly used model of human cDCs [26]. Making use of S6, a SIRT6 inhibitor that our group has recently identified (compound 5 in the original study [23]), we evaluated the effects of SIRT6 blockage on moDC differentiation and immunostimulatory capacity. In line with the effects of Sirt6 deletion in BMDCs, inhibiting SIRT6 in differentiating moDCs was found to prevent both the downregulation of CD14 and the expression of CD1a which typically occur in response to GM-CSF and IL-4 (Figure 6A, B) [26], with the strongest effects observed at the highest S6 concentration. Consistent with the ability of S6 to block monocyte differentiation into moDCs, cells that had been cultured in the presence of the SIRT6 inhibitor also exhibited a markedly reduced capacity to stimulate the proliferation of allogeneic lymphocytes in MLR (Figure 6B). Thus, these data confirmed that SIRT6 plays a crucial role in ensuring proper differentiation and function of human cDCs, too.

## DISCUSSION

In this work, we addressed for the first time the role of Sirt6 in lineage determination, maturation and function of cDCs, defining a new function for Sirt6 within the immune system that complements those connected to the control of cytokine expression already described elsewhere [13, 15-18].

Our study reveals that Sirt6 promotes cDC differentiation and maturation at different levels. In the first place, we were able to demonstrate reduced frequencies of CMPs and of cDC precursors in the BM of Sirt6KO mice compared to WT animals, indicating that the role of Sirt6 in cDC generation is relevant *in vivo*. Notably, pDCs were found to be similarly represented in WT and Sirt6KO BMs, indicating a specific role of Sirt6 in the cDC lineage. MEP were also expressed at lower frequencies in BMs from Sirt6KO animals, while differentiation into the granulocyte-macrophage lineage appeared to be enhanced (as judged based on the frequency of GMP, granulocytes and monocytes/macrophages in Sirt6KO BMs).

Our data clearly indicate that *in vitro* BMDC generation is inefficient when Sirt6 is lacking. Such impairment in BMDC generation, as well as, likely, the reduced representation of cDC precursors in Sirt6KO BMs could be explained by the reduced availability of TNF-α, a cytokine that contributes to BMDC differentiation and maturation [2, 24, 25] and whose secretion is compromised in Sirt6KO cells [13, 15, 16] (including BM cells). Notably, the increased myelogranulocytic hematopoiesis found in Sirt6KO mice is also in line with a lack of TNF-α and consequent removal of TNF-α-mediated inhibition of colony-forming units-granulocyte [30]. Supplementation of Sirt6KO BMDC cultures with concentrations of TNF-α that were similar to those found in WT BMDCs (0.5-1 ng/ml) did increase the frequency of conventional CD11c^+^ cells recovered. However, the rescue effect we observed was partial. In fact, higher concentrations of TNF-α (10-20 ng/ml) even reduced the recovery of Sirt6KO BMDCs, which could reflect the tendency of high amounts of TNF-α to switch CD34^+^ progenitor differentiation towards the macrophage lineage [31]. The immuno-phenotype of these BM cultures (CD11c^−^CD11b^+^LyC^+^LyG^−^, data not shown) indeed supports this notion. On the one hand, these results support a role for a reduced availability of TNF-α in the less efficient generation of BMDCs that was typically observed with Sirt6KO BMs [24, 25]. On the other hand, the impossibility to fully revert this phenotype suggests the involvement of additional mechanisms. For instance, IL-6 was shown to inhibit cDC differentiation and maturation *in vivo* in mice [32]. Among the different potential cellular sources of this cytokine, mesenchymal stem cells were reported to produce high IL-6 amounts, thereby preventing cDC differentiation [33]. We observed increased IL-6 production in mesenchymal stem cells generated from Sirt6KO mice (D.L., unpublished data). In addition, Sirt6KO BMDCs (both in baseline conditions and, even more, in response to TLR ligands) were found to produce high quantities of this cytokine. Thus, increased IL-6 secretion by cDC precursors and by cells from the microenvironment (i.e. mesenchymal stem cells) could both contribute to reduce cDC representation in the BM of Sirt6KO mice and to reduce the yield of BMDCs generated *in vitro*.

In addition to a role for Sirt6 in cDC differentiation, our data also indicate a key function for this deacetylase in cDC maturation. Namely, both the spontaneous maturation process that BMDCs cultured with GM-CSF undergo and BMDC maturation that occurs in response to TLR ligands were defective in the absence of Sirt6. Sirt6KO BMDCs cultured in the absence of TLR ligands exhibited a reduction in the frequency of both immature CD11c^+^MHCII^low^CD86^low/neg^ and mature CD11c^+^MHCII^high^CD86^high^ BMDCs, and a preferential accumulation of immature CD11c^+^MHCII^−^CD86^−^ BMDC precursors. Notably, a more immature phenotype of the Sirt6KO BMDCs was confirmed by an increased propensity to perform endocytosis (a property that is typical of immature cDCs and that is downregulated during maturation) and by a reduced immunostimulatory capacity in MLR [1, 26]. Given the reduced TNF-α concentrations detected in Sirt6KO BMDC cultures and the established role of this cytokine in promoting cDC maturation [1, 26], we assessed the effect of TNF-α supplementation on this phenotype. A restoration of the frequency of mature BMDCs could be achieved at the highest TNF-α concentrations used (10-20 ng/ml), while the same conditions essentially failed to lead to a representation of immature BMDCs within Sirt6KO cell preparations that would be comparable with the one observed in WT BMDCs. These data suggest that Sirt6's ability to regulate TNF-α secretion is involved in the final maturation of BMDCs. However, again, the contribution of additional mechanisms cannot be excluded. TLR ligands, such as LPS and CpG, were used to test the ability of Sirt6KO BMDCs to undergo a full maturation [1]. These stimuli did induce MHCII, CD80, CD86, and CD40 upregulation both in WT and in Sirt6KO cDC. However, the expression of these markers was typically much lower in Sirt6KO BMDCs compared to WT BMDCs. Notably, CCR7 expression in Sirt6KO BMDCs was also markedly reduced, both in baseline conditions and after stimulation with TLR ligands. Consistently, CCL21-induced migration was reduced in Sirt6-deficient BMDCs. These observations are relevant as they show for the first time a contribution of Sirt6 to the expression of costimulatory molecules, including CD40, and of CCR7 in cDCs, as well as, consequently, to CCL21-driven cDC migration.

Cytokine expression by cDCs is typically fine-tuned and is essential for the priming of T cell responses, but also for the termination of inflammatory responses and for self-tolerance maintenance [1]. Skewed cytokine release by cDCs may result in immune defects and/or autoimmunity. Our study highlights differential effects of Sirt6 deletion on cytokine production by BMDCs. In baseline conditions (in the absence of TLR ligands), Sirt6 depletion was found to result in a reduced expression of TNF-α and IL-12, while IL-6 production was increased. On the other hand, Sirt6KO BMDCs showed an increase propensity to secrete TNF-α and IL-6 in response to TLR ligands, with the exception of CpG, which binds TLR9 and that was previously shown to stimulate the production of lower amounts of cytokines by Sirt6KO cells [15, 16]. Higher TNF-α and IL-6 production in response to TLR ligands in Sirt6KO cells could reflect increased NF-κB and AP-1 activity in these cells, as suggested by previous reports [18, 19]. Notably, in the case of TNF-α production in response to TLR ligands, the latter mechanisms appear to prevail on Sirt6's ability to promote the secretion of this cytokine by direct deacylation [14], which, in itself, would rather explain a reduction, instead of an increase, in TNF-α secretion by Sirt6KO BMDCs (which indeed occurs, but only in unstimulated BMDCs). On the other hand, the molecular basis for the apparently selective role of Sirt6 in mediating TLR9-dependent cytokine production remains to be defined. The qPCRs we performed to monitor the levels of *Tlr9* and of *Unc93b1*, a protein with a key role in the trafficking of endosomal TLRs [28, 29], failed to detect significant differences between Sirt6KO and WT BMDCs. Moreover, Sirt6KO BMDCs responded with an increased TNF-α and IL-6 production in response to TLR ligands that bind other TLRs localized within the endosomes, such as TLR3 and TLR7/8, which essentially rules out a major defect in the uptake of TLR ligands due to Sirt6 deletion. In line with this notion, endocytosis was rather increased, instead of reduced, in Sirt6KO BMDCs. Interestingly, IL-12 production was found to be subjected to a completely different regulation by Sirt6, in that, in Sirt6KO BMDCs, it was uniformly defective in response to all of the stimuli used. These data demonstrate for the first time a crucial role for Sirt6 in IL-12 regulation. Moreover, overall, they confirm that the effects of Sirt6 on cytokine production can be very different, depending on the cytokine and on the type of stimulus that cells encounter.

Here we also took advantage of the availability of a recently identified SIRT6 inhibitor (S6) [23] to study the role of SIRT6 in human moDCs. Using this tool, we were able to find a striking effect of SIRT6 inhibition in this cDC model, in that monocyte differentiation into moDCs was virtually completely blocked by the SIRT6 inhibitor as judged based on CD14 downregulation and CD1a upregulation, both of which did not occur when SIRT6 was inhibited. Consistent with a major defect in moDC differentiation, cells generated in the presence of the SIRT6 inhibitor failed to stimulate allogeneic lymphocytes in MLR. Thus, these data essentially confirm that SIRT6 plays a critical role in human cDC differentiation and function, too.

In aged humans, lower percentages of circulating cDCs are found in the peripheral blood [5] and aged cDCs lose their ability to migrate to the lymph nodes and to prime CD4^+^ and CD8^+^ T cells [6]. In addition, consistent with the patterns of circulating cytokines found in elderly and that were shown contribute to frailty, morbidity and mortality [34, 35], cDCs from aged subjects typically secrete excessive amounts of IL-6 and TNF-α [4, 6]. Given that Sirt6 expression and function probably decrease during aging [10-12], it is appealing to speculate that such a reduction in Sirt6 activity may contribute to determine these features of aged cDCs, and, thereby, play a causative role in immunosenescence [36].

In conclusion, we propose a key role for Sirt6 in cDC differentiation, maturation and function. We show that Sirt6 controls cDC lineage commitment *in vivo* and *in vitro*, acting very early during myeloid differentiation in a way which is only partially dependent on its ability to promote TNF-α production. In fact, our data strongly suggest that other mechanisms in addition to TNF-α secretion, including, possibly, Sirt6's activity as an epigenetic regulator of NF-κB [19, 37], are likely to contribute to its roles in cDCs. Our data clearly indicate a role for Sirt6 in the expression of costimulatory molecules, including CD40, as well as in the production of IL-12. Besides, we also provide evidences for a role for this deacetylase in regulating CCR7 expression and, as a result, CCL21-induced cDC migration. Overall, Sirt6 emerges from this study as a master regulator of cDC biology. This work opens new scenarios for future investigations of the complex epigenetic mechanisms involved in the regulation of cDC function.

## MATERIALS AND METHODS

### Animals and hematology

129/Sv Sirt6^+/−^ mice (2-8 month old) were bred weekly to generate WT and Sirt6KO mice. Mice were kept in temperature- and light-controlled conditions and fed *ad libitum*. All experiments were performed in accordance with the relevant laws and institutional guidelines for animal care and use following approval by the Institutional Animal Care and Use Committee of the IRCCS A.O.U. San Martino-IST (Genoa, Italy). In order to avoid the potentially confounding effects of the acute metabolic syndrome that affects these mice in their fourth week of life, we performed all the immunological tests and isolated bone marrow (see below) using 16-18-days old Sirt6KO mice (or aged-matched WT littermates). At this age, no difference in the percentage of apoptotic immune cells between WT and Sirt6KO mice could be detected *ex vivo* by standard Annexin-V/propidium iodide staining and flow cytometry analysis of cells isolated from the thymus (data not shown). For blood counts determination, ~100μl blood were collected in EDTA coated tubes and blood counts were determined using an automated analyser (scil Vet abc Plus).

### BMDC and moDC generation and stimulation

Following mouse BM harvest by flushing of tibiae and femurs, BMDCs were generated by culturing total bone marrow cells at 1 × 10^6^cells/ml density in RPMI-1640 supplemented with 10% Hyclone^®^ serum, 100 U/ml penicillin-streptomycin, 5 × 10^−2^ mM β-mercapto-ethanol, 1% not essential amino acids, 1% sodium pyruvate, containing 20 ng/ml GM-CSF (Carlsbad, CA, USA) [21, 22]. At day 6, cells were harvested and reseeded in fresh medium in presence of 5 ng/ml GM-CSF. BMDC differentiation was detected by monitoring CD11c expression. BMDC maturation was induced by stimulation with the following TLR ligands: 100 ng/ml LPS [Sigma Aldrich, 0111:B4 serotype; TLR4 ligand], 1 μg/ml CpG (1826; Tib Molbiol; TLR9 ligand), 1 μg/ml R848 (Enzo Life Sciences; TLR7/TLR8 ligand); 50 μg/ml Zym (Sigma Aldrich; TLR2, dectin 1, dectin 2 ligand), or 25 μg/ml polyI:C (Enzo Life Sciences; TLR3 ligand). For the detection of cytokine concentrations in cell supernatant, 2 × 10^5^ BMDCs were seeded in 96-wells and stimulated for 24 h with or without TLR ligands (see above). For immunophenotyping and cytokine detection by intracellular staining, BMDCs were harvested at day 8 of culture and 3 × 10^6^ BMDCs were plated in 12-well plates and stimulated with or without TLR ligands at the previously mentioned concentrations in presence of 1 μg/ml Brefeldin A (BFA, Sigma Aldrich). In the rescue experiments with TNF-α, total BM cells or BMDCs harvested at day 6 of culture were cultured in presence of increasing concentrations (0.5, 1, 10, or 20 ng/ml) of mouse recombinant TNF-α (Miltenyi Biotech GmbH).

moDCs were generated from adherent monocytes as described previously [26, 38]. In brief, buffy coat preparations from healthy volunteers were obtained from the blood bank of the IRCCS A.O.U. San Martino-IST (Genoa, Italy). Peripheral blood mononuclear cells (PBMCs) were isolated by Ficoll-Paque (Biochrom) density gradient centrifugation. Cells were resuspended in serum-free X-VIVO 20 medium (Cambrex) and seeded (1 × 10^7^ cells/well) for 2 h in 6-well plates at 37°C. Afterward, non-adherent cells were removed by extensive washing with PBS (Life Technologies). moDCs were generated by culturing the adherent monocytes in RPMI-based medium supplemented with 100 ng/ml GM-CSF (molgramostim, Leucomax) and 20 ng/ml human recombinant IL-4 (R&D) in the presence or absence of S6 [compound 5 from [23]; purchased from Asinex] at the indicated concentrations, or of vehicle DMSO. At day 6 of culture, cells were harvested for subsequent immuno-phenotyping and used as stimulators in MLR.

### Immunostaining and flow cytometry

The monoclonal antibodies (mAbs) that were used for the immunophenotyping and for intracellular cytokine staining in BMDCs were the following: CD11c (clone G418), MHCII (I^A^/I^E^, clone M5/114.15.2), CD86 (clone GL1), CD80 (clone 16-10A1), CD40 FITC (clone 3/23), CCR7 (CD197, clone 4B12), TNF-α (clone MP6-XT22), IL-6 (clone MP5-20F3) were all from Biolegend (CA, USA). Anti-MHCI (clone 28.8.6), and anti-IL-12 (p40/p70, clone C15.6) were from Becton Dickinson (BD; NJ, USA). The mAbs that were used for the *ex vivo* immunophenotyping of BM cells and of BMDCs were the following: Ter119 (clone TER119, BD), CD19 (clone 1D3, BD), CD3 (clone 17A2 Biolegend), CD8a (clone 53-6.7, Biologend), CD4 (clone GK1.5, Biolegend), Gr1 (clone RB6-8C5, Biolegend), B220 (clone RA3-6B2, Biolegend), CD16/CD32 (clone 2.4G2, BD), CD34 (clone HM34, Biolegend), CD117 (c-kit, clone ACK2, Life Technologies), Sca-1 (clone D7, Biolegend), CD127 (IL7R clone LG.7F9, Ebioscience), Ly6C (clone HK1-4, Biolegend), Ly6G (clone 1A8, Miltenyi Biotech), CD11b (clone M1/70, Biolegend), PDCA1 (clone JF05-1C2.4.1, Miltenyi Biotech). Within BM cells, the combined expression of Gr1 and CD11b was used to discriminate between granulocytes (Gr1^+^CD11b^+^) and the mono/macrophage subpopulation (Gr1^−^CD11b^+^). pDCs were identified as CD11c^int^CD11b^−^PDCA1^+^B220^+^ cells. CD34^+^ progenitors were characterized as lin^−^Sca1^−^CD117^+^. A subsequent analysis of the combined expression of CD16/CD32 and CD34 was performed to discriminate between granulocyte/monocyte progenitors (GMP, CD16/CD32^high^CD34^+^), common myeloid progenitors (CMP, CD16/CD32^low^CD34^+^) and megakaryocyte/erytrocyte progenitors (MEP, CD16/CD32^low^CD34^−^). Common lymphoid progenitors (CLP) were identified as lin-Sca1-CD117^+^CD127^+^ cells. Cell were stained in FACS buffer [PBS with 1% fetal calf serum (FCS)] for 15 min at 4°C, washed and a minimum of 30.000 cells/sample were analyzed by flow cytometry. Intracellular cytokine detection was done according to the instructions of the manufacturers. In brief, BMDCs were stained with mAbs against surface markers, fixed and permeabilized using cytofix/cytoperm kit^™^ (BD). Cells were subsequently incubated with mAbs for cytokines and, following extensive washings, they were analyzed by flow cytometry. Human moDC immunophenotyping was performed with mAbs against CD14 (BD) and CD1a (Dako). All the flow cytometry analyses were done on a FACScanto™ cytometer and analyzed using Flow-Jo_V10 software (Tree Star) or CellQuest.

### Dextran uptake detection

Quantitative analysis of endocytosis was performed as described elsewhere [39]. In brief, 2 × 10^5^ BMDCs harvested at day 7 of culture were incubated with FITC-dextran (MW 40,000, Invitrogen) for 30 min at 37°C or at 4°C. Endocytosis was blocked by 2 washes in ice-cold PBS with 1% FCS. Cells were then stained for CD11c alone or in combination with MHCII and subsequently analyzed by flow cytometry with a FACScanto™ cytometer.

### ELISAs

TNF-α, IL-6 and IL-12 levels in cell supernatants were evaluated by commercially available ELISAs (Biolegend, California) according to the manufacturer's instructions.

### MLR

For the MLRs utilizing mouse cells, 5 × 10^5^ CFSE (2 μM)-labeled allogeneic (Balb-C, H-2K^D^) CD4^+^ cells (responders) were cultured with BMDCs (stimulators) from WT or Sirt6KO mice that were harvested at day 8 of culture and γ-irradiated (25 Gy). Cells were plated in U-bottomed 96-well microplates (Nunc) at S:R ratios of 1:5, 1:10, 1:100, and 1:1,000. Proliferation of alive (propidium-iodide negative) CD3^+^CD4^+^CD11c^−^ cells was evaluated by CFSE dilution after a 5-day incubation. As a positive control, CFSE dilution in response to 5 μg/ml PHA was utilized. For the MLRs utilizing human cells, 10^5^ allogeneic PBMCs cells (responders) were cultured in 96-well U-bottomed microplates with 10^4^ stimulator cells (moDCs). Proliferation was measured on day 5 with ATPlite^TM^ (Perkin Elmer) according to the instructions of the manufacturer.

### Chemotaxis assays

BMDC Chemotaxis was evaluated by a transwell approach. Briefly, 1 × 10^5^ LPS-pretreated (overnight)-BMDCs (harvested at day 6) were seeded in the upper chamber of 96-well transwell chambers and allowed to migrate through a polycarbonate mesh (pore size: 5 μm) at 37°C. CCL21 (100 ng/ml; Biolegend, CA, USA) or medium alone (as a control for spontaneous migration) were placed in the lower chamber to assess CCR7-dependent chemotaxis. After 3 h, cells that had migrated to the lower chamber (150 μl) were counted by acquiring a fixed volume with an Attune® cytometer. Data were analyzed with the Attune® Software v2.1.0. CCL21-dependent migration was calculated by subtracting the number of cells that had spontaneously migrated. Each experiment was performed in duplicate. Values are given as percentage of migrated cells and normalized to WT (100%).

### qPCR

Total RNA was extracted from cells using the RNeasy minikit (Qiagen S.r.l., Milan, Italy) according to the manufacturer's instructions. 1 μg of RNA was reverse transcribed in a final volume of 50 μl using the High Capacity cDNA Reverse Transcription kit (Invitrogen). 5 μl of the resulting cDNA was used for qPCR with a 7900 HT Fast real-time PCR system (Applied Biosystems by Invitrogen) using GoTaq® qPCR Master Mix (containing Sybr® green dye) and specific primers for mouse *Tlr2 Tlr3*, *Tlr7*, *Tlr8*, *Unc93b1*. All the data obtained were normalized to *Hprt1* housekeeping gene expression.

### Statistical analyses

Data are presented as means ± SEM. T-tests were used to assess statistical differences between two experimental groups. Graphpad prism 5.0 software was used for all of the analyses.

## SUPPLEMENTARY MATERIAL FIGURES AND TABLE


