# Supplementary material for: Sirt6 regulates dendritic cell differentiation, maturation, and function
Source: Aging (Albany NY). 2016 Jan 12;8(1):34–47. doi: 10.18632/aging.100870 (PMC4761712; doi:10.18632/aging.100870)
Supplement: Supplementary file 1 [file aging-08-034-s001.pdf]

SUPPLEMENTARY FIGURES

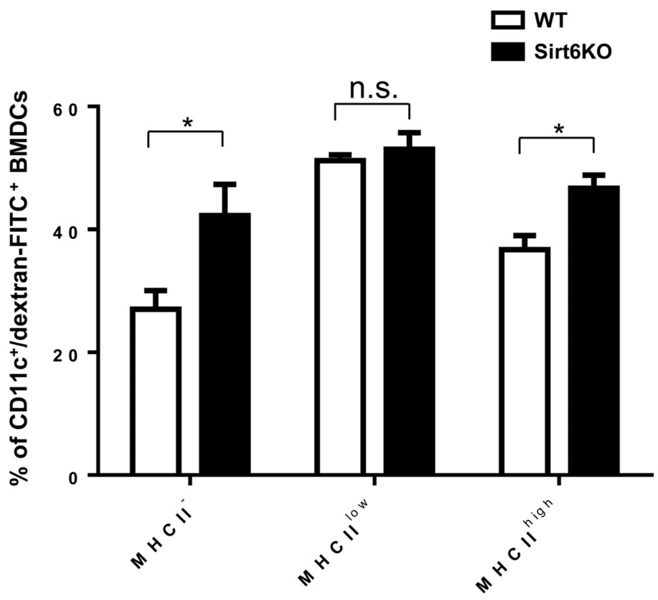

Supplementary Figure 1. Sirt6KO BMDC precursors and mature BMDCs exhibit an increased endocytic activity. WT and Sirt6KO BMDCs were harvested at day 7 and incubated with dextran-FITC for 30 min at 37°C. Subsequently, cells were stained for CD11c and MHCII and finally analyzed by flow cytometry. Results are means ± SEM of two separate experiments, n=3 for each genotype.

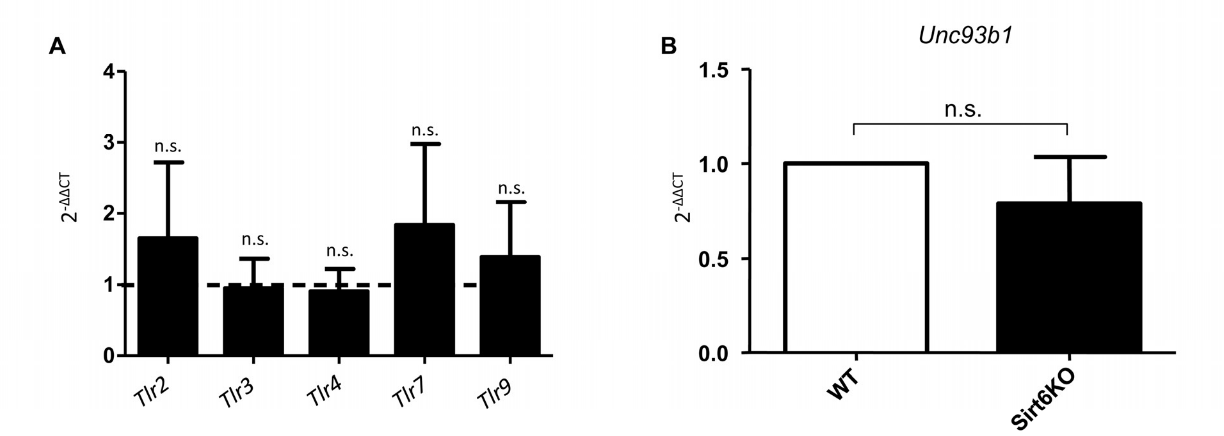

Supplementary Figure 2. Effect of Sirt6 deletion on *Tlrs* and *Unc93b1* expression. (**A**, **B**) WT and Sirt6KO BMDCs were harvested at day 7 and the expression of *Tlr2, Tlr3, Tlr4,Tlr7,Tlr9* and *Unc93b1* was analyzed by qPCR. Results are mean ± SEM of seven (**A**) or three (**B**) separate experiments (n=3-7 for each genotype).

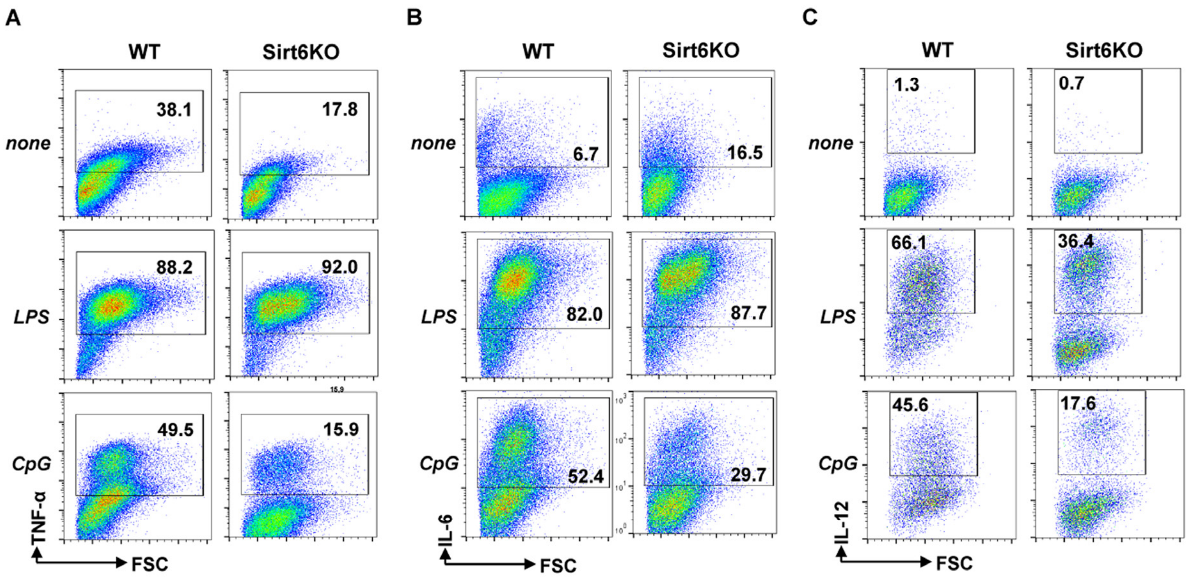

Supplementary Figure 3. Sirt6 deletion skews TNF-α, IL-6 and IL-12 production in BMDCs. (**A-C**) WT and Sirt6KO BMDCs were harvested at day 8 and stimulated for 24 h with or without LPS or CpG. Thereafter, cells were harvested, and, following staining for intracellular TNF-α, IL-6, or IL-12, they were analyzed by flow cytometry. One representative experiment out of five is presented, n=3-10 for each genotype.

Blood test	WT	Sirt6KO
White blood cell count (x10^3 cells/μL)	5.4±0.6	4.4±1.7$^{n.s}$
Red blood cell count (x10^6 cells /μL)	6.5±0.4	6.0±1.0$^{n.s}$
Hemoglobin (g/dL)	11.4±0.8	9.8±2.6$^{n.s}$
Hematocrit (%)	40.5±3.3	36.5±8.8$^{n.s}$
Mean corpuscular volume (fL)	62.3±2.5	60.8±7.4$^{n.s}$
Mean corpuscular hemoglobin (pg)	17.6±0.8	16.2±2.2$^{n.s}$
Mean corpuscular hemoglobin concentration (g/dL)	28.2±0.6	26.7±0.9*
Platelet count (x10^3/μL)	724.3±119	441.3±99*

Supplementary Table 1. Hematology profile of WT and Sirt6KO mice (d17-18). 100 μL blood was collected from 17d/18d-old WT (n=4) or Sirt6KO (n=4) mice and blood counts were determined with an automated hematology analyzer. n.s.: not significant; *: p<0.05.
